# BCG Substrains Change Their Outermost Surface as a Function of Growth Media

**DOI:** 10.3390/vaccines10010040

**Published:** 2021-12-29

**Authors:** Sandra Guallar-Garrido, Farners Almiñana-Rapún, Víctor Campo-Pérez, Eduard Torrents, Marina Luquin, Esther Julián

**Affiliations:** 1 Departament de Genètica i de Microbiologia, Facultat de Biociències, Universitat Autònoma de Barcelona, Bellaterra, 08193 Barcelona, Spain; sandra.guallar@uab.cat (S.G.-G.); far_almy@hotmail.com (F.A.-R.); victor.campo@uab.cat (V.C.-P.); marina.luquin@uab.cat (M.L.); 2 Bacterial Infections and Antimicrobial Therapies Group, Institute for Bioengineering of Catalonia (IBEC), Baldiri Reixac 15-21, 08028 Barcelona, Spain; etorrents@ibecbarcelona.eu; 3 Microbiology Section, Department of Genetics, Microbiology and Statistics, Biology Faculty, Universitat de Barcelona, 08028 Barcelona, Spain

**Keywords:** cell wall, hydrophobicity, lipid, neutral red, PDIM, PGL

## Abstract

*Mycobacterium bovis* bacillus Calmette-Guérin (BCG) efficacy as an immunotherapy tool can be influenced by the genetic background or immune status of the treated population and by the BCG substrain used. BCG comprises several substrains with genetic differences that elicit diverse phenotypic characteristics. Moreover, modifications of phenotypic characteristics can be influenced by culture conditions. However, several culture media formulations are used worldwide to produce BCG. To elucidate the influence of growth conditions on BCG characteristics, five different substrains were grown on two culture media, and the lipidic profile and physico-chemical properties were evaluated. Our results show that each BCG substrain displays a variety of lipidic profiles on the outermost surface depending on the growth conditions. These modifications lead to a breadth of hydrophobicity patterns and a different ability to reduce neutral red dye within the same BCG substrain, suggesting the influence of BCG growth conditions on the interaction between BCG cells and host cells.

## 1. Introduction

One hundred years after being developed, *Mycobacterium bovis* bacillus Calmette-Guerin (BCG) is still the preferred prophylactic option for preventing tuberculosis (TB) worldwide. BCG vaccine effectiveness appears to be influenced by several factors including the genetic background and/or immune status of the vaccinated population, BCG substrain used as a vaccine, interference with environmental nontuberculous mycobacteria, etc. [1]. The same paradigm is found when BCG is used to treat bladder cancer (BC) patients, in which approximately 25–30% of them are unresponsive to BCG.

To understand the variability of efficacy, a comparison of BCG vaccines needs to be performed using exactly the same parameters through in vitro, ex vivo, and in vivo experiments and in clinical trials to obtain reliable and comparable results. Currently, BCG vaccines comprise several substrains that present genetic differences [1] and, consequently, different phenotypic characteristics. However, modifications to phenotypic characteristics can also be influenced by culture conditions [2]. Accordingly, mycobacteria are prone to lose the ability to synthesize cell wall lipids, which are costly and *unnecessary* in vitro.

To obtain high BCG production, BCG is traditionally grown as a pellicle on the surface of Sauton liquid medium. Cells are then collected, suspended in an aqueous solution, and lyophilized. After several drawbacks such as the loss of viability due to aggressive pellicle disruption [3], freeze-drying steps, or the difficulty of ensuring reproducibility among batches, the WHO established protocols for standardizing BCG production [4]. However, other parameters, such as the specific compositions of the culture media, are not systematically regulated. Subsequently, different formulations for BCG growth are currently used.

A proper comparison among BCG substrains is essential for TB or BC, and BCG is also a plausible tool for the treatment of many immunological disorders, different cancers [5], and diabetes, as well as nonspecific protection against a broad spectrum of pathogens by inducing trained immunity [6]. Thus, the influence of culture conditions on BCG characteristics needs to be elucidated more than ever. Here, we focused our research on mycobacterial superficial characteristics, because they govern host immune system–mycobacteria interactions. We evaluated the presence of lipids such as phthiocerol dimycocerosates (PDIMs) or phenol glycolipid (PGL), which are distinctively expressed among BCG substrains [7,8], physicochemical properties such as hydrophobicity, and the neutral red reducing power of five different BCG substrains grown on two Sauton formulations currently used for BCG production.

## 2. Materials and Methods

### 2.1. Bacterial Strains and Culture Conditions

The BCG substrains Phipps (ATCC 35744) and Tice (ATCC 27289) strains were purchased from the Spanish Type Culture Collection and from the German Collection of Microorganisms and Cell Cultures, respectively. BCG Moreau, BCG Pasteur, and BCG Glaxo were kindly given to us by Dr. Carlos Martin (Zaragoza, Spain), Dr. Maria Jesús García (Madrid, Spain), and by Dr. Jun Liu (Toronto, ON, Canada), respectively. All BCG strains were maintained on Middlebrook 7H10 agar (Difco Laboratories, Surrey, UK) supplemented with 10% oleic-albumin-dextrose-catalase enrichment medium at 37 °C. A suspension of each BCG substrain in phosphate-buffer saline was prepared, and 0.3 mL of the suspension containing 1.5 × 10^6^ CFU was carefully added to the surface of each liquid medium. Then, BCG substrains were cultured as pellicles under static conditions in glass bottles containing two formulations of Sauton medium (asparagine plus 60 mL/L glycerol (A60) and glutamate plus 15 mL/L glycerol (G15)) as previously described [9] for 4 weeks at 37 °C. Macroscopic appearance is shown in Supplementary Figure S1. *Escherichia coli* (ATCC 25922), used as a negative control for hydrophobicity experiments, was cultured in Luria-Bertani medium for one day at 37 °C.

### 2.2. Extraction of Superficial Non-Covalently Linked Lipids

To extract superficial lipids from BCG pellicles obtained after static incubation, 20 mL of petroleum ether (40–60°, PE) was added to the top of the pellicle and rested for 5 min. Then, this PE was recovered, evaporated and analyzed by thin-layer chromatography (TLC). TLC plates were eluted with PE (60–80 °C):diethyl ether (90:10, *v*/*v*) or chloroform:methanol (96:4, *v*/*v*) and were revealed by spraying with 10% molybdophosphoric acid in ethanol and heating the plates at 120 °C [10]. Three independent pellicles for each condition were analyzed.

### 2.3. Neutral Red Staining

Neutral red staining was performed on mycobacteria obtained from three independent pellicles for each condition, as previously described [11].

### 2.4. BCG Hydrophobicity by Hexadecane-Aqueous Buffer Partitioning

BCG pellicles were filtered, dried, and transferred to glass tubes, and two washes with phosphate urea magnesium sulphate (PUM) buffer were performed [12]. Then, 3 mL of the suspensions were transferred to a new tube containing 2.4 mL of hexadecane vortexed and incubated at 37 °C for 8 min. After 15 min at room temperature (RT), the bottom phase was recovered, and the OD was measured at 400 nm (Infinite 100 PRO, Tecan, Switzerland). The percentage of hydrophobicity was obtained by comparing the OD values of the bacterial suspension with or without hexadecane. Three independent pellicles for each condition were analyzed.

### 2.5. BCG RNA Isolation and Quantitative Real-Time PCR (qRT-PCR) Assay

BCG cell pellets were resuspended in 300 μL of TE buffer (10 mM Tris-HCl, 1 mM EDTA, pH 8) plus lysozyme (0.4 g/mL). After 5 min at RT, 300 μL of lysis buffer plus β-mercaptoethanol (20 μL/mL) (Sigma-Aldrich, St. Louis, MI, USA) and glass beads (150–200 μm) were added to the tubes. These tubes were heavily shaken through four cycles of 50 s in a rotor stator homogenizer (IKA, Staufen, Germany), and RNA extraction was performed following the GeneJET RNA Purification Kit (Thermo Fisher Scientific, Madrid, Spain). The isolated RNA was treated with 10xTURBO DNase (Life Technologies, Carlsbad, CA, USA), quantified and reverse-transcribed to cDNA using Maxima Reverse Transcriptase (Thermo Fisher Scientific) along with random hexamer primers (Thermo Fisher Scientific). Expression of genes (*papA5* and *ppsA*) involved in the production of PDIM/PGL, and gene *Rv0577* responsible for CFP32 protein synthesis, were analyzed by qRT-PCR using PowerUp™ SYBR™ Green Master Mix (Applied Biosystems, Foster City, CA, USA) in a StepOnePlus™ Real-Time PCR System (Applied Biosystems) according to the manufacturer’s protocol applying a comparative Ct (ΔΔCt) as quantification method. The primers used in all qRT-PCRs are listed in Supplementary Table S1, results were normalized using the expression of 16S rRNA housekeeping gene. Two independent pellicles for each condition were analyzed.

### 2.6. Statistical Analysis

All experiments were performed with at least two (RT-PCR analysis) or three (lipids and hydrophobicity studies) different cultures of each BCG substrain. The hydrophobicity experimental results were analyzed using an ANOVA test, respectively, with GraphPad Prism 9.0 software (San Diego, CA, USA). The data represent the mean ± standard deviation (SD). *p* < 0.05 was considered significant.

## 3. Results

### 3.1. The Outermost Lipids and Hydrophobicity of the BCG Cell Wall Depend on the Growth Conditions

**Figure 1:**
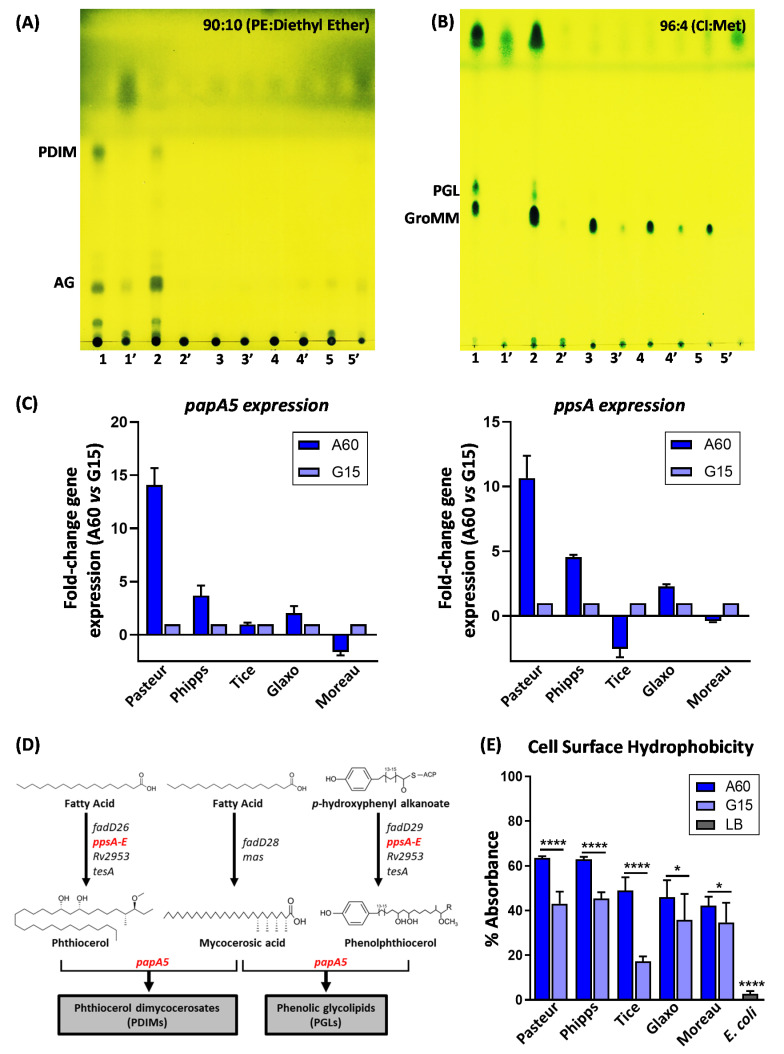
Analyses of the outermost surface of BCG substrains. (**A**,**B**) Thin-layer chromatography (TLC) corresponding to the superficial lipids extracted using petroleum ether (PE) at 40–60 °C for 5 min of BCG substrain pellicles grown on A60 and G15 Sauton media. The volume of lipid extracts applied to TLC plates was equal under all conditions and derived from the same pellicle surface. The results of one representative experiment out of at least three biological replicates. TLC elution was performed (**A**) in PE:diethyl ether at 90:10 (*v*/*v*) or (**B**) in chloroform:methanol at 96:4 (*v*/*v*). Both plates were treated with 10% molybdatophosphoric acid in ethanol. BCG-Pasteur (Lines 1 and 1′); BCG-Phipps (Lines 2 and 2′); BCG-Tice (Lines 3 and 3′); BCG-Glaxo (Lines 4 and 4′); and BCG-Moreau (Lines 5 and 5′). 1-5 BCG substrains grown on A60 Sauton medium and 1′-5′ BCG substrains grown on G15 Sauton medium. (**C**) Fold ratio of *papA5* and *ppsA* expression in BCG substrains grown on A60 medium relative to that of BCG substrains grown on G15 medium. (**D**) Scheme of genes involved in PDIM and PGL synthesis. (**E**) Affinity of BCG substrains towards hexadecane, *E. coli* was used as a negative control. The results are expressed as a percentage of the initial absorbance at 400 nm. appearance. Data represent the mean ± standard deviation (SD) from three independent experiments. * *p* < 0.05, **** *p* <0.0001 (ANOVA test).

As shown by TLC (Figure 1A,B and Table 1), BCG-Pasteur and BCG-Phipps, which synthesize PDIM and PGL when cultured in 7H10 [8], also produced these lipids when grown on A60. When the expression of genes involved in the synthesis of PDIM and PGL was assessed [13], the lipid analysis correlated with a significantly increased expression of *ppsA* and *papA5* genes in both strains grown on A60 compared with those grown on G15 medium (Figure 1C,D). However, no traces of PDIM were observed on the BCG-Tice surface when growing in any Sauton composition, despite producing PDIM when cultured on 7H10. BCG-Glaxo was unable to produce PDIM or PGL (Figure 1A,B) although high expression of both genes involved in their synthesis was observed (Figure 1C). As previously discussed [14], some still unknown genetic lesions could be responsible for the PDIM/PGL defect in BCG-Glaxo. As expected, BCG-Moreau was unable to produce either PDIM or PGL in any culture media. The deletion of 975 bp that affects in part *ppsA* in the BCG-Moreau strain leads to PDIM/PGL defects [15]. Additionally, BCG substrains produced glycerol monomycolate (GroMM) only when cultured on A60. Remarkably, the presence of GroMM, PDIM, and PGL on the mycobacterial surface was diminished when mycobacteria grew on G15 compared to their presence on A60. As Figure 1E shows, the cell hydrophobicity increased with increasing glycerol concentration in the culture media. In all cases, cells grown on A60 showed the highest hydrophobicity.

### 3.2. Culture Media Composition Influences BCG Neutral Red Staining

**Figure 2:**
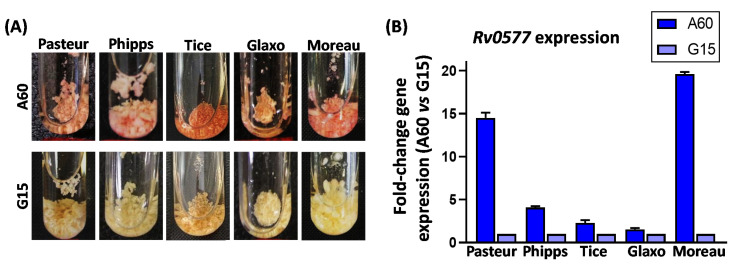
Neutral red staining and *Rv0577* gene expression of BCG substrains. (**A**) Images corresponding to neutral red staining in cells of BCG substrains grown on different culture media. (**B**) Fold change of *Rv0577* expression in BCG substrains grown on A60 medium relative to that of BCG substrains grown on G15 medium. Two independent pellicles for each condition were analyzed.

When the neutral red dye is prepared in barbital buffer, a color shift from yellow to red is observed in the presence of compounds able to transfer protons to the dye. All tested BCG substrains grown on A60 immediately adopted an intense red coloration, in contrast to the yellowish coloration taken on BCG substrains grown on G15 (Figure 2A). This result was correlated with a higher expression of the *Rv0577* gene, encoding CFP32, in all BCG substrains grown on A60 compared with those grown on G15 (Figure 2B).

## 4. Discussion

Our results demonstrate that culture media can modify the PDIM/PGL expression, cell wall hydrophobicity, and the neutral red staining of all tested BCG substrains (Pasteur, Phipps, Tice, Glaxo, and Moreau). The lipidic profile of BCG substrains is well known when grown on 7H10 medium [8,16], but the usual production of BCG for clinical applications is performed in Sauton media [9,17]. Here, we describe for the first time a rapid loss of these lipid molecules by BCG after only a shift in culture medium from Middlebrook to Sauton [8]. Previous studies pointed out that consecutive subcultures of *M. tuberculosis* in Middlebrook medium tended to increase PDIM-depleted mycobacteria [18], hinting that in vitro cultures could favor the loss of these lipids, which are not necessary for in vitro survival, while maximizing mycobacterial growth. Moreover, the immunostimulatory lipid GroMM [19] was produced only in glycerol-rich media, showing high expression under increasing glycerol concentrations in culture medium [20]. Similar to the relevant role of PDIM/PGL described in *M. tuberculosis*, the loss of PDIM and PGL in BCG-Pasteur has been associated with a reduction in virulence and protective efficacy against TB [7]. Thus, the same substrain grown under different conditions could trigger different immunomodulatory activities, which are relevant in vaccine efficacy, as demonstrated in a murine model by Venkataswamy and coworkers [21]. In the case of BC, recent studies in our laboratory demonstrated in vitro [9] and in vivo [22] different antitumour effects of BCG-Connaught grown on different media, which could also be related to the superficial characteristics expressed.

Our results show that the culture medium also modified mycobacterial surface hydrophobicity. BCG substrains grown on G15 showed significantly less hydrophobicity than those grown on A60, and these observed differences may account for the diminished abundance of lipids. Because BCG-Tice was unable to synthesize PDIM when growing in both Sauton media, GroMM was responsible for the high hydrophobicity. Cell hydrophobicity is related to mycobacterial clump formation, which makes it difficult to achieve a homogeneous suspension in aqueous solution, hindering mycobacteria-host interactions and diminishing BCG efficacy in both TB and BC treatments [17]. Moreover, the necessity of BCG hydrophobicity for TB aerosol vaccination has been recently considered [23].

Additionally, neutral red staining was different among BCG substrains grown on the different culture media. Neutral-red positivity implies the presence of some compounds able to transfer protons to the dye and has been related to virulence in *M. tuberculosis* [11]. Alterations in the lipidic content of the *M. tuberculosis* cell wall such as the loss of more than one lipid-containing polymethyl-branched fatty acid [24] lead to a negative neutral-red reaction. Here, we observed that all BCG substrains grown on G15 were neutral-red negative, coinciding with those devoid of lipids in the outermost layer. In addition, BCG strains able to produce either PDIM and PGL or a high content of GroMM showed an intense red coloration. Altogether, these results indicate that in BCG, as in *M. tuberculosis*, the response to neutral red staining could be related to lipid profile modifications, although it cannot be directly attributed to these neutral lipids [24]. Cell wall lipids may confer a structure that allows the entrance of the dye and/or the exposition of other molecules with reducing power capability. CFP32, a protein exclusive to the *M. tuberculosis* complex [25], which can be either secreted or present in different mycobacterial cell subfractions, has been related to neutral red staining. The biological function of CFP32 is found in the methylglyoxal detoxification pathway, which is related to glycerol metabolism. Accordingly, *M. tuberculosis* overproducing CFP32 multiplied 25% faster than the parental strain grown in glycerol-containing medium, suggesting that CFP32 might participate in the detoxification of glycerol metabolism by-products [26]. In our experiments, BCG cells grown on A60 showed the highest red intensity and higher *Rv0577* gene expression, which could be related to the abundance of the encoded protein CFP32 [27]. Interestingly, CFP32 regulates the immune response by interacting with TLR2 [28], which is associated with BCG immunogenicity. Further research is thus needed to clarify the role of this molecule in BCG immunomodulatory capability.

## 5. Conclusions

One hundred years after BCG development, we are still trying to understand the protective effect against TB disease and the mechanism of BCG in preventing bladder cancer recurrence and progression. Altogether, our results demonstrate that different growth culture media lead to specific outermost surface profiles, providing BCG substrains with specific physico-chemical characteristics that can modify their immunogenicity. Therefore, not only host factors as a variable for BCG efficacy but also BCG culture conditions should be considered.

## Figures and Tables

**Table 1:** Superficial lipidic pattern of BCG substrains (Pasteur, Phipps, Tice, Glaxo, and Moreau) grown on two different Sauton compositions (A60 and G15) that differ by amino acid source, L-asparagine (A) or L-glutamate (G), and the glycerol concentration, 60 mL/L (60) or 15 mL/L (15). PDIM: phthiocerol dimycocerosate; AG: acylglycerol; PGL: phenolic glycolipid; GroMM: glycerol monomycolate. +, present; ++, highly present; +++, abundantly present; -, not present.

	Culture Media	BCG Pasteur	BCG Phipps	BCG Tice	BCG Glaxo	BCG Moreau
**PDIM**	A60	+++	++	-	-	-
	G15	-	-	-	-	-
**AG**	A60	+++	+++	-	-	+
	G15	+	-	-	-	+
**PGL**	A60	++	+	-	-	-
	G15	-	-	-	-	-
**GroMM**	A60	+++	+++	++	++	++
	G15	-	-	+	+	-

## Data Availability

The data presented in this study are available on request from the corresponding author.

## Supplementary Materials

The following supporting information can be downloaded at: https://www.mdpi.com/article/10.3390/vaccines10010040/s1, Figure S1: Macroscopic appearance of BCG pellicles grown on different culture media; Table S1: List of primers used for Quantitative Real-Time PCR (qRT-PCR) Assay.
