# Supplementary material for: BCG Substrains Change Their Outermost Surface as a Function of Growth Media
Source: Vaccines (Basel). 2021 Dec 29;10(1):40. doi: 10.3390/vaccines10010040 (PMC8779077; doi:10.3390/vaccines10010040)
Supplement: Supplementary file 1 [file vaccines-10-00040-s001.zip › vaccines-1461209-supplementary.pdf]

vaccines MDPI

Supplementary Materials:

The following are available online at https://www.mdpi.com/article/10.3390/vaccines10010040/s1, Figure S1: Macroscopic appearance of BCG substrains cultures grown on Sauton media, Table S1: List of primers used in qRT-PCR assays.

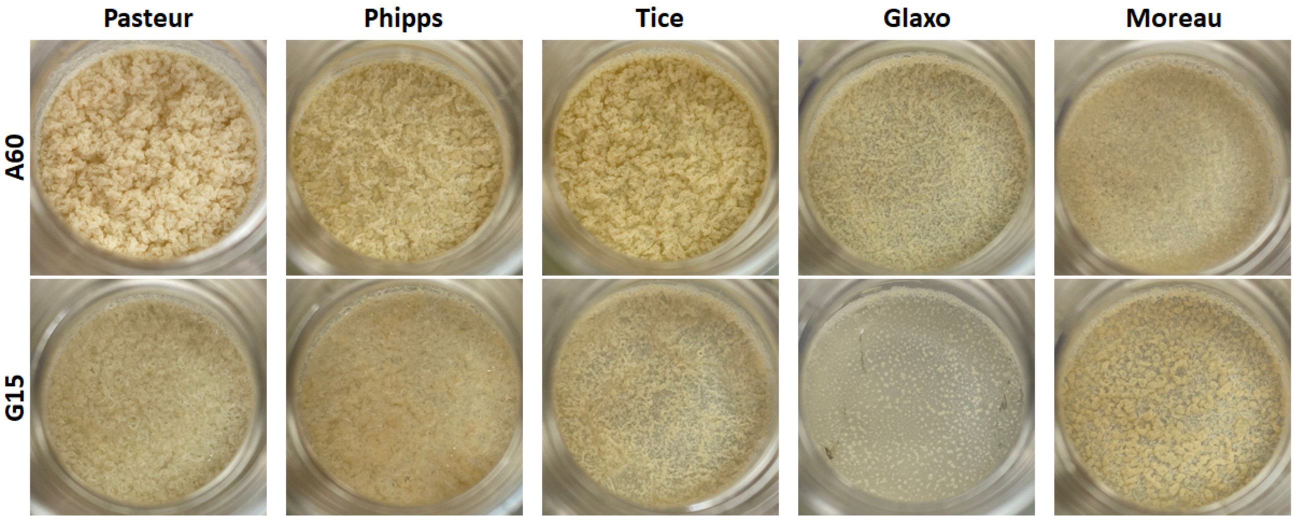

Figure S1. Macroscopic appearance of BCG substrains cultures grown on two Sauton media (A60 and G15).

Table S1. List of primers used in qRT-PCR assays.

Primer name	Sequence 5´-3´	Observations
***Rv0577*_Fw**	CTTCAGACCACCGATCAGTC	Putative glyoxylase CFP32: Protein involved in the methylglyoxal detoxification pathway. Induces maturation of dendritic cells in a TLR2-dependent manner. Induces Th1-polarized immune responses.
***Rv0577*_Rv**	GTTGTCGTCGTAACCCCAG	
***papA5*_Fw**	CTTGGAAACCCACCCAGTC	Polyketide-associated protein (Pap): Acetyltransferase involved in the PDIM and PGL synthesis, catalyzes the diesterification of phenolphthiocerols with mycocerosates.
***papA5*_Rv**	GGCAACGAGATTCCAACCG	
***ppsA*_Fw**	GGCATGGGATGTCGTTTCC	Phthiocerol polyketide synthase subunit A: Part of the *Pps*ABCDE complex involved in the biosynthesis of the lipid core common to phthiocerols and phenolphthiocerols
***ppsA*_Rv**	GGCGTTCACAGAGAAAGTCC	
***16S rRNA*_Fw**	CAACGCGAAGAACCTTACCT	Housekeeping gene: Used as a constitutive expression control, useful for normalizing the expression level of genes of interest among samples.
***16S rRNA*_Rv**	TGCACACAGGCCACAAGGGA	
